# Differences in Anticipatory Behaviour between Rats (*Rattus norvegicus*) Housed in Standard versus Semi-Naturalistic Laboratory Environments

**DOI:** 10.1371/journal.pone.0147595

**Published:** 2016-01-28

**Authors:** I. Joanna Makowska, Daniel M. Weary

**Affiliations:** Animal Welfare Program, University of British Columbia, Vancouver, British Columbia, Canada; ETH Zurich, SWITZERLAND

## Abstract

Laboratory rats are usually kept in relatively small cages, but research has shown that they prefer larger and more complex environments. The physiological, neurological and health effects of standard laboratory housing are well established, but fewer studies have addressed the sustained emotional impact of a standard cage environment. One method of assessing affective states in animals is to look at the animals’ anticipatory behaviour between the presentation of a cue signalling the arrival of a reward and the arrival of that reward. The primary aim of this study was to use anticipatory behaviour to assess the affective state experienced by female rats a) reared and housed long-term in a standard laboratory cage versus a semi-naturalistic environment, and b) before and after treatment with an antidepressant or an anxiolytic. A secondary aim was to add to the literature on anticipatory behaviour by describing and comparing the frequency and duration of individual elements of anticipatory behaviour displayed by rats reared in these two systems. In all experiments, total behavioural frequency was higher in standard-housed rats compared to rats from the semi-naturalistic condition, suggesting that standard-housed rats were more sensitive to rewards and experiencing poorer welfare than rats reared in the semi-naturalistic environment. What rats did in anticipation of the reward also differed between housing treatments, with standard-housed rats mostly rearing and rats from the semi-naturalistic condition mostly sitting facing the direction of the upcoming treat. Drug interventions had no effect on the quantity or form of anticipatory behaviour, suggesting that the poorer welfare experienced by standard-housed rats was not analogous to depression or anxiety, or alternatively that the drug interventions were ineffective. This study adds to mounting evidence that standard laboratory housing for rats compromises rat welfare, and provides further scientific support for recommendations that current minimum standards be raised.

## Introduction

In the wild, Norway rats (*Rattus norvegicus*) engage in a host of behaviours, from foraging and building burrows to traveling several kilometers per day patrolling their territory [1,2]. Rats are inquisitive and motivated to explore their surroundings [3–6]. In a laboratory, the descendants of the Norway rat are typically kept in relatively small cages with few stimuli to explore and few opportunities to perform behaviours other than sleeping and reaching for food and water. Research has shown that laboratory rats prefer larger and more complex environments [7–12], and when they are placed in a semi-natural environment, they display a behavioural repertoire similar to that of their wild relatives [13–15].

The physiological [16], neurological [17] and health [18,19] effects of current laboratory housing standards are well established, but fewer studies have addressed the sustained emotional impact of a standard cage environment. An animal’s emotional well-being is central to the concept of animal welfare [20] and new advances in animal welfare science have given rise to a variety of methods of studying affective states in animals [21]. Some studies have assessed the affective consequences of housing rats in an ‘enriched’ environment (what this involves varies widely across studies), but the control group is often housed in conditions that can be considered worse [22–24] or better [25,26] than the common Canadian standard system that houses two rats with a piece of PVC pipe. Moreover, few studies have evaluated females, and fewer still have evaluated animals reared in standard versus enriched systems for longer than a few weeks.

One method of assessing affective states in animals consists of looking at their anticipatory behaviour–that is, the behaviour exhibited in the interval between a signal of the impending arrival of a reward and the arrival of that reward. Research has suggested that the level of anticipatory behaviour, usually measured as total behavioural frequency, displayed by an animal is influenced by the animal’s underlying affective state, suggesting that differences in anticipatory behaviour can be used to make inferences about animal welfare [27,28]. It has been shown that ‘impoverished’ animals exhibit a stronger anticipatory response than ‘normal’ animals, and that severely depressed (i.e. anhedonic) animals fail to show an anticipatory response altogether. For example, male rats housed in standard laboratory cages exhibited more anticipation before access to a sucrose solution than rats housed in enriched cages [26]. Male rats subjected to a long-term severe stressor (social defeat followed by months of isolation) did not display an anticipatory response before access to sucrose [29], but treatment with an antidepressant restored the anticipatory response [30]. Thus, there appears to be a curvilinear relationship between affective state and anticipatory behaviour: poor welfare is associated with increased anticipation, but in extreme cases, anhedonia may reverse the more typical relationship. When two groups of animals differ in their level of anticipation, testing after administration of a mood-enhancing drug (e.g. antidepressant) offers a way to explore the nature of the affective state experienced by each group.

It is well documented in humans and other animals that exposure to stressors affects sensitivity to rewards at a behavioural and neurophysiological level [31–33]. Individuals deprived of essential stimuli are more sensitive not only to the particular stimuli they are deprived of, but to all rewarding and aversive stimuli [34,35]. Spruijt et al. [36] proposed that differences in the level of anticipation reflect differences in reward sensitivity, which in turn are related to an animal’s subjective evaluation of his or her internal state and environment [27]. Reward sensitivity is mediated by the opioid and dopaminergic systems, and the display of anticipatory behaviour is the result of the release of endorphins and dopamine. In general, the release of endorphins or dopamine causes increased locomotor activity, and this change in behaviour can facilitate the finding of resources [36,37]. Indeed, Spruijt et al. [36] argue that the ‘characteristic’ behavioural pattern exhibited in anticipation of a reward resembles the behavioural pattern induced by the injection of a low dose of opioids.

However, there seem to be no accounts in the literature describing what ‘characteristic’ anticipatory behaviour looks like. When it was first written about, anticipatory behaviour was described generally as a “state of agitation” that manifests externally as “restlessness” or “activity” [38]. More recently, anticipatory behaviour has been characterized as an increased level of activity resulting from frequent and abrupt transitions between short fragments of behaviour [36,39]. One study on rats reported that the most frequent behavioural categories exhibited by standard-housed rats anticipating a reward were exploration, locomotion and arousal [39]. Descriptions of individual behaviours, and descriptions for rats housed in non-standard cages, do not seem to have been published.

The primary aim of this study was to use anticipatory behaviour to assess the affective state experienced by female rats a) reared and housed long-term in standard laboratory cages versus a semi-naturalistic environment, and b) before and after treatment with an antidepressant or an anxiolytic. A secondary aim was to add to the literature on anticipatory behaviour by describing and comparing the frequency and duration of individual elements of anticipatory behaviour displayed by rats reared in these two systems.

Antidepressants such as selective serotonin reuptake inhibitors (SSRIs) need to be taken for several weeks before they are clinically effective [40], but ketamine given at low doses is effective within hours (e.g. review in humans: [41]; studies in rats: [42–44]). For this reason ketamine was the antidepressant selected for this study. Traditional anxiolytics cause sedation, which would not be appropriate when studying behavioural activation. Therefore, we used α-S1 tryptic casein, a non-sedating, naturally derived protein used in veterinary medicine to treat anxiety in cats, dogs and horses. This protein is effective in rats, with anxiolytic effects similar to those seen with a medium dose of a benzodiazepine [45–47]. Doses selected for this study were within the range known to be effective in similar studies but without causing behavioural activation or suppression.

## Materials and Methods

### Ethics statement

This work was approved by the University of British Columbia’s Animal Care Committee (protocol number: A12-0179). All procedures were performed in accordance with the Canadian Council on Animal Care guidelines on care and use of rodents in research.

### Animals and housing

Forty-two, 22-to 23-day-old female Sprague-Dawley rats were purchased from Charles River Laboratories Canada. As soon as they arrived at our facility, they were systematically assigned to either standard cages (n = 6 cages; two rats per cage) or semi-naturalistic cages (n = 6 cages; five rats per cage). In assigning rats to housing treatment, the experimenter alternated between standard and semi-naturalistic cages, and within each cage alternated between rats huddled at the back of the shipping box and those who reared at the front.

All cages were in one room, and cage type was symmetrically distributed across the room. Rats were housed under a reversed light cycle, with lights off from 10:00–22:00 h so that all testing was performed during rats’ active period. During training and testing, the room was illuminated with a low pressure sodium light (Master SOX-E 18W, Royal Philips, Amsterdam, the Netherlands) that emits yellow-orange light visible to humans but likely not to rodents [48]. Temperature and humidity were kept at (mean ± SD) 23.3 ± 0.8°C and 36 ± 15%, respectively.

Rats were marked once by means of a spot applied to their coat with a permanent nontoxic animal marker (Stoelting Co., Wood Dale, IL, USA) for individual identification. Rats took part in a year-long, observational study before being used here. Training for the experiments described here began when rats were 14 months old, and testing began when rats were 19 months and weighed (mean ± SD) 636 ± 56 g (n = 8) in the standard cages and 541 ± 68 g (n = 20) in the semi-naturalistic cages. Rats were 21 months old at the end of the last experiment and weighed 672 ± 69 g (n = 7) in the standard cages and 577 ± 72 g (n = 20) in the semi-naturalistic cages. Morbidity and mortality were expected given the long-term nature of treatments. Rats developing health problems, which consisted of a tumour in approximately three quarters of cases of morbidity in each housing condition, were removed from the study. At the beginning of testing there remained eight standard-housed rats (four cages each housing two rats), and 20 semi-naturalistic-housed rats (six cages each housing two (n = 2), three (n = 2) or five (n = 2) rats).

Standard cages were made of polycarbonate and measured 45 x 24 x 20 cm (L x W x H); they were fitted with a wire lid and a filter top (Ancare Corp., Bellmore, NY, USA) to minimize the transmission of smells and sounds from the semi-naturalistic cages. Each cage contained aspen chip bedding (Northeastern Products Corp., Warrensburg, NY, USA), a piece of PVC pipe (approximately 18-cm in length and 10-cm in diameter) and two pieces of brown paper towel. Once behavioural training and testing began, rats were housed in a standard cage that had an 8-cm diameter opening drilled into one end; this opening was covered from the outside with a piece of Plexiglas held with industrial strength Velcro. This opening allowed us to connect the standard cage to a testing cage without having to handle the rats before testing. Rats had *ad libitum* access to rat chow (LabDiet^®^ 5012, PMI^®^ Nutrition International, LLC, Brentwood, MO, USA) and tap water. Cages were cleaned and rebedded twice a week by the facility’s animal care technician.

The semi-naturalistic cages (Critter Nation^™^ double unit with stand, MidWest Homes for Pets, Muncie, IN, USA) measured 91 x 64 x 125 cm (L x W x H). They were made of horizontal galvanized wire bars that allowed climbing, and offered four levels (lined with removable plastic inserts) connected by ramps. The lower portion of each cage was lined with Plexiglas, which allowed us to fill the bottom 30-cm of the cage with a mixture of black earth, compost, and sphagnum peat moss (3-in-1 Landscape Soil, Premier LiteWay, Rivière-du-Loup, QC, Canada). This soil substrate was watered every few days to prevent it from drying out and causing burrows to collapse [49]. Burrow construction and maintenance caused soil to fall outside the cage, so fresh soil was added as needed to maintain levels. Each cage contained two litter boxes (filled with aspen chip bedding), several pieces of PVC tubing, a hammock, a lava rock, and a horizontal rope across the top floor. On occasion, rats were also provided with timothy hay or strips of paper that they could access by pulling through the wire bars or removing from a PVC tube; rats typically used these items to line their hammock. The top shelf was lined with polar fleece blankets that rats could burrow into.

Semi-naturalistic-housed rats also had *ad libitum* access to rat chow and tap water, but their diet was supplemented three to five times per week with various types of unsweetened cereal, nuts, seeds or oats usually provided in a large bowl and mixed with clean aspen chip bedding, so that rats had to sort through the wood chips to find the treats. Once a week, the PVC tubes and plastic inserts lining each level were removed and disinfected (Quatricide^®^ PV, Pharmacal Research Laboratories, Inc., Waterbury, CT, USA), litter boxes were changed, and fleece blankets were laundered. Plastic inserts were wiped down (Mohawk FloorCare Essentials, CHEMSPEC, Baltimore, MD, USA) every second day between washing. These tasks were performed by a laboratory assistant, and occasionally by the experimenter.

All rats approached the experimenter’s hand when it was placed in their cage. However, unlike the standard-housed rats who were handled twice a week during cage changing, rats housed in the semi-naturalistic environment were rarely handled because they always chose to retreat into a burrow rather than to be picked up. For this reason, experiments were designed to avoid handling rats before testing.

### Pilot Study: Individual anticipatory behaviour

In the Pilot Study, anticipation of a sweet food reward (Honey Nut Cheerios^®^, General Mills Canada Corporation, Mississauga, ON, Canada) was tested individually in an arena that was similar in size for rats from both housing conditions; there were no drug interventions.

#### Testing apparatus

**Fig 1:**
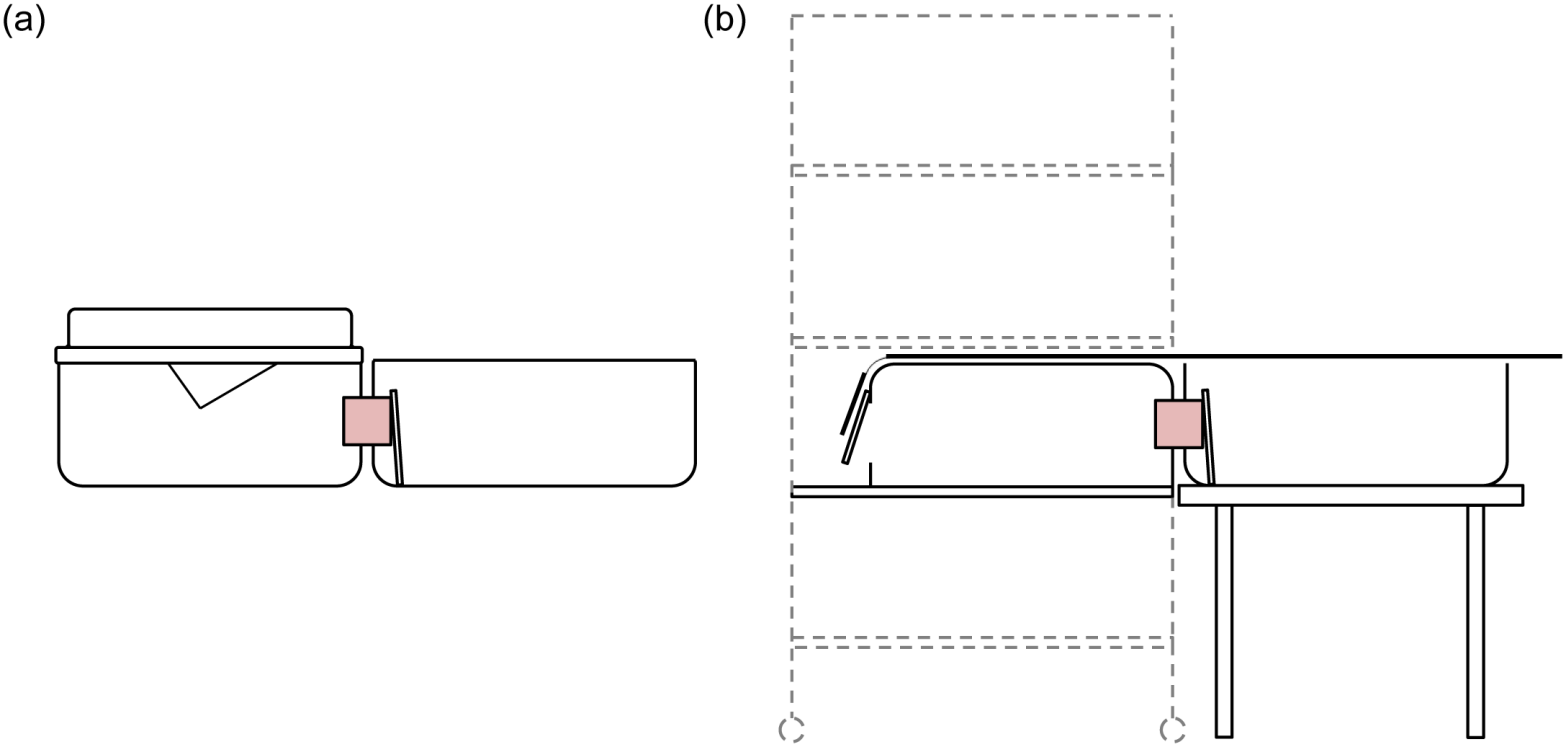
Testing apparatus used in the Pilot Study for rats housed in standard (a) and semi-naturalistic (b)cages. In both cases, the treat cage (48 x 38 x 20 cm) is on the right and is connected to the home cage via a red transparent tunnel (7.6 cm diameter x 7.7 cm long). Tunnel exit into the treat cage was blocked with a piece of Plexiglas during 60 s cue-reward interval. For semi-naturalistic-housed rats, an inverted standard cage was placed inside the home cage. One end of this inverted cage connected to the red tunnel while the other side had a hole (10 cm diameter) covered from the outside with an oversized piece of Plexiglas (‘flap door’) on hinges. A rope system allowed the experimenter to open the flap door when a rat approached, allowing her to enter. This way, rats could not enter unless let in by the experimenter, but once inside, they could exit by pushing on the flap door from the inside. Only one rat was allowed inside at a time.

The testing apparatus consisted of the rats’ home cage connected to a treat cage via a short tunnel (Fig 1). To enable us to test rats individually and in the same space as standard-housed rats without having to handle them, the testing apparatus for rats housed in the semi-naturalistic environment also included an inverted standard cage that was placed inside the semi-naturalistic cage, on the bottom shelf. All testing equipment was cleaned between cages.

#### Testing procedure

The procedure for standard cages was as follows: The experimenter removed the filter top, water bottle, wire lid and PVC pipe, and gently picked up the rat not being tested and placed her in a holding cage containing familiar bedding. The home cage lid and filter top were placed back, the piece of Plexiglas covering the hole drilled into one side of the home cage was removed, and the cage was joined to the treat cage via the tunnel. The tunnel exit was blocked with a piece of Plexiglas. The experimenter delivered the conditioned stimulus (three ‘beep’ sounds from a Timex^®^ Triathlon digital stopwatch) and stood to the left of the home cage. After 60 s, the Plexiglas barrier was removed and the rat could access 14 reward items in the treat cage. The rat in the holding cage remained there while her companion ran the trial; after the first rat completed her trial, roles were reversed. Order of testing alternated between trials.

The procedure for semi-naturalistic cages was as follows: The experimenter opened a door at the front of the cage and placed the inverted standard cage on the bottom shelf. One end of this inverted cage had a flap door (see Fig 1) and the other end was connected to the treat cage via the tunnel; the tunnel exit was blocked with a piece of Plexiglas. As soon as a rat approached the flap door, this door was pulled open by the experimenter and the rat could enter the inverted cage. Only one rat was allowed to enter the cage at a time. The experimenter immediately delivered the conditioned stimulus and stood to the left of the home cage. After 60 s, the Plexiglas barrier was removed and the rat could access the reward items in the treat cage. Once a rat ate all the reward items, the flap door was opened and the rat exited.

Testing alternated between standard and semi-naturalistic cages. Cages were tested in the same order every day, and each cage was tested once a day at the same time each day to control for daily rhythmic differences in activity.

Rats from both housing treatments had to be trained daily over several weeks to cross the tunnel and retrieve treats from the treat cage. Anticipatory behaviour training took place once a day, as lag time between sound cue and access to the treat cage was gradually increased from 0 to 60 s. Rats were trained to the behavioural criterion of 60 s, and this took 7–14 days depending on the individual. Training and testing were performed in the animals’ housing room.

#### Data collection

All rats were initially trained to perform this experiment, but not all were willing to participate. One standard-housed rat never acclimated to the attached treat cage and was excluded from the study due to persistent burying of the tunnel leading to the treat cage. Only 12/20 semi-naturalistic-housed rats entered the inverted cage, and of those only five were willing to remain for the required 60 s. Therefore, sample size for the Pilot Study was seven standard-housed rats from four cages and five semi-naturalistic-housed rats from five cages.

All trials were recorded directly onto a laptop using a high definition webcam (Microsoft^®^ LifeCam Studio 1425, Redmond, WA, USA; 30 frames/s). Standard-housed rats all reached the 60-s criterion on the same day, but data were only collected on this first day for those rats in each pair who ran the trial first (i.e. were not placed in the holding cage before their trial) and on the second day for the remaining standard-housed rats, when order of testing was reversed. For consistency, data were also collected on the first or second day a semi-naturalistic-housed rat reached the 60 s criterion, except for one semi-naturalistic-housed rat whose initial videos were poor quality so we scored the third day. Therefore, data were collected on the first day rats reached the 60-s criterion for four standard- and three semi-naturalistic-housed rats, on the second day for three standard- and one semi-naturalistic-housed rat, and on the third day for one semi-naturalistic-housed rat.

Videos were scored using The Observer XT 9.0 (v.9.0.436, Noldus Information Technology, Wageningen, the Netherlands). Behaviours were scored using an ethogram adapted from Draper [50] and van der Harst et al. [26] (Table 1). During data collection we noticed that rats would spend long periods of time rearing, but this rearing was not static. Indeed, rats would often shift positions–their front paws would go from leaning on one wall to leaning on another–without touching the ground in between. We believe that these shifts in position while already rearing reflect behavioural activity and should be captured in the measure of total behavioural frequency, and therefore scored them as ‘rear-move’, versus ‘rear-only’ for the initial rear. In addition, we were interested in the location of rats’ focus when they were sitting; therefore, the behaviour ‘sit’ was further qualified as ‘sit-treat’ in which rats sat facing the location where the treat would appear (in this case, sitting with their head in the tunnel), and ‘sit-only’ in which rats sat facing any other direction.

**Table 1:** Ethogram used in the Pilot Study and in Experiments 1–2.

Agonistic behaviour*	Two or more rats engaged in offensive or defensive behaviour; pinning or being pinned down, pawing at each other, gripping skin
Alert	Head raised suddenly, body and head held still, body appears tense
Bite	Biting on the wire lid or wire bars
Climb	Rat is suspended vertically with all four paws on a vertical surface
Dig	Rapid, successive movements of the front and/or back paws while displacing bedding or dirt
Drink	Rapid licking at the spout of the water bottle
Eat	Rat is pawing at the food hopper in an attempt to grab rat chow, or eating something she picked up
Groom self	Maintenance behaviours; includes face washing, coat cleaning, and scratching
Groom (social)*	Licking or nibbling of fur by or of a conspecific
Jump	Rat bends down before springing up, with four paws momentarily in the air at once
Lie down	Rat’s abdomen is resting on a flat surface; body is not supported by the paws
Mounting	Placing of forequarters over the hindquarters of a conspecific, or inspecting/submitting to anogenital inspection (lordosis)
Rear	Upper body is raised, with front paws either unsupported or resting on a vertical surface
Rear-only	Rat rears after performing some other behaviour
Rear-move	Starting in a rear position, rat moves both front paws into a new position
Shake	Quick shake of entire body
Sit	All paws and hind quarters on the ground, no forward locomotion; rat may be looking around or pivot without moving hind paws
Sit-only	Rat sits facing any direction other than the location of upcoming treat
Sit-treat	Rat sits facing the location of upcoming treat (Pilot Study: head in the red tunnel; Exp. 1 & 2: experimenter)
Sniff (non-social)	Sniffing air, ground or object; air: head raised and slightly pointing upwards with minor up-down movements; ground or object: nose contacting the ground or object
Sniff (social)*	Rat’s nose contacts another rat; excludes anogenital inspection
Stretch	Rat elongates her limbs and abdomen and arches her back
Sway	Rat is standing still except for slow left-right movements of the head
Turn	While remaining in the sitting position, rat turns around to face a different direction
Urination	Rat lifts her hind quarters and the base of her tail, holds still for a few seconds
Walk	Forward locomotion, often includes sniffing of the ground; all four paws are moving
Yawn	Rat briefly opens mouth wide
Out of sight	Rat is partially or fully out of view, precluding observation

Asterisks denote social behaviours, which were only scored in Experiments 1 and 2.

#### Statistical analysis

Data were analysed using the SAS software (v.9.3). Because visual inspection of residuals revealed that data were not normally distributed and not amenable to transformation, and because parametric statistics are non-robust for small sample sizes, we used the non-parametric Mann-Whitney U test to compare total behavioural frequency and frequency and duration of individual behaviours between standard and semi-naturalistic cages. Most behaviours never occurred or occurred very rarely, so only rear-only, rear-move, sit, sit-only, sit-treat and walk were analysed statistically. For comparisons of duration, rear-only and rear-move were combined into a single category called ‘rear’. For data presentation purposes, durations were converted into percent trial time. All *p* values are two-tailed.

### Experiments 1 and 2: cage-level anticipatory behaviour with drug treatment

Results from the Pilot Study revealed that a major change in methods was required; the testing procedure in the Pilot Study resulted in a small sample size (many rats avoided the testing apparatus; see Data Collection section above), biased sampling (only the boldest individuals were likely included) and a different relationship with the testing apparatus for rats from the two housing conditions (inclusion of the inverted cage inside the semi-naturalistic cages). To avoid these problems, in Experiments 1 and 2 anticipation of a sweet food reward (slice of ripe banana, 3-mm thick) was tested directly in the home cage (and therefore in the presence of cage mates). Each rat was tested at baseline, under the influence of an antidepressant (Exp. 1) or an anxiolytic (Exp. 2), and after return to baseline (Exp. 1).

#### Testing procedure

The experimenter delivered a sound cue (three ‘beep’ sounds from a digital stopwatch) and stood motionless to the left of the home cage. After 300 s, each rat was given one slice of banana. For standard cages, the filter top was removed for the duration of the trial.

Cages were tested in the same order every day, alternating between standard and semi-naturalistic cages. Each cage was tested twice per day (one morning and one afternoon trial) at the same time of day. Rats were tested twice in each condition to give them the opportunity to learn through experience the new incentive value of the reward once they were in a new (drug-induced) motivational state [51]. Although all rats in the cage participated in the anticipatory task at every trial, each day data were collected only from one rat per cage per drug treatment (baseline vs. on drug vs. back to baseline). The order in which individuals from each cage were observed was determined at random.

In Experiment 1, the drug intervention was an antidepressant (ketamine hydrochloride, Bioniche Animal Health Canada Inc., Belleville, ON, Canada; 42 mg/kg). In Experiment 2, the drug intervention was a nutritional supplement with anxiolytic properties (α-S1 tryptic casein, Vétoquinol N.-A. Inc., Lavaltrie, QC, Canada; 15 mg/kg). Drugs were delivered in a treat: the appropriate amount of drug was mixed with one teaspoon of peanut butter with honey (Kraft Canada Inc., Don Mills, ON, Canada) and sandwiched between two crackers (Ritz Munchables Buttery Thins, Christie Brown & Co., Mississauga, ON, Canada). All rats received a peanut butter cracker sandwich at the same time, but only the target rat from each cage received a sandwich laced with the drug. This rat was observed closely to ensure that in no case was the sandwich hoarded or stolen by a cage-mate.

In Experiment 1, rats were trained over 11 days to associate the sound cue with arrival of a slice of banana in this context (some rats had already learned the association between this sound cue and the delivery of a treat in the Pilot Study); lag time between sound cue and reward was gradually increased from 5 to 300 s, usually in 30 s increments. After training was complete, all cages were tested daily until data collection was complete. Three weeks after the completion of Experiment 1, rats were re-trained on the anticipatory task over three trials. Then, each cage was tested daily until data collection was complete. Training and testing were performed in the animals’ housing room. Timelines for testing in these two experiments are presented in Tables 2 and 3.

**Table 2:** Timeline for Experiment 1.

Day	AM trial	+ 3 hrs	PM trial	+ 1 hr	sandwich
**1**	baseline		baseline		regular
**2**	(not recorded)		(not recorded)		antidepressant
**3**	on drug		on drug		regular
**4–10**	(not recorded)		(not recorded)		regular
**11**	back to baseline		back to baseline		regular

**Table 3:** Timeline for Experiment 2.

Day	AM trial	+ 2 hrs	sandwich	+ 1 hr	PM trial	+ 1 hr	sandwich
**1**	(not recorded)		regular		baseline		anxiolytic
**2**	(not recorded)		anxiolytic		on drug		regular

Rats were tested twice daily and given a regular or drugged peanut butter cracker sandwich between the two daily trials (Exp. 2 only) and at the end of the day (Exp. 1 and 2). Baseline activity was collected on what counted as Day 1 for a particular rat; activity on the drug was collected on Day 2 or 3 (Exp. 2 and 1, respectively); and return to baseline was collected on Day 11 (Exp. 1).

#### Data collection

In Experiment 1, all but two rats from the semi-naturalistic housing condition were tested; these two rats were excluded because one did not eat the ketamine sandwich and the other developed a health problem. Therefore, sample size for Experiment 1 was eight rats from four standard cages, and 18 rats from six semi-naturalistic cages. One standard-housed rat had to be euthanized for humane reasons after her data collection was complete, so the ‘back to baseline’ data was collected only from her now singly housed cage-mate after she had become single-housed. This single-housed rat was included in the analysis because her results were within the range of results we obtained from her when she was pair-housed.

In Experiment 2, only two rats per cage were tested (for cages housing more than two rats, subjects were chosen at random), but three rats from the semi-naturalistic housing condition were excluded from analysis because consumption of the entire sandwich (i.e. full drug dose) could not be confirmed. Therefore, sample size for Experiment 2 was seven rats from four standard cages, and nine rats from five semi-naturalistic cages.

Trials were video recorded with a high definition camcorder (Canon HD10, Japan; 25 frames per s) and scored using The Observer XT 9.0. The same ethogram was used as in the Pilot Study but with the addition of several social behaviours (Table 1). In these experiments, sitting orientation (sit-only versus sit-treat) was not obvious in the standard cages, so standard-housed rats were simply scored as ‘sit’ without further qualification. Scoring was from video with the scorer blind to drug treatment and time of day, but not housing condition (the latter was impossible given that testing was in the home cage).

#### Statistical analysis

Residuals were examined to verify normality and homogeneity of variances. In Experiment 1, paired sample t-tests revealed no differences in the total frequency, or frequency or duration of individual behaviours, between the morning and afternoon trials, so data were averaged to obtain one value per rat per day. Most behaviours occurred rarely, so the effect of drug treatment, cage type, and their interaction on the frequency and duration of these behaviours were not analysed statistically. The effect of drug treatment, cage type, and their interaction on total behavioural frequency, as well as frequency and duration of rear-only, rear-move, sit, walk and groom self were analysed using a mixed model in SAS (v.9.3) following [52]. For tests of duration, rear-only and rear-move were analysed as a single category called ‘rear’. The model included rat and cage as random effects, drug treatment as a repeated measure, and the Kenward-Roger degrees of freedom approximation to account for unbalanced data (i.e. different number of rats per cage and cages per housing treatment). We also included a contrast statement in the mixed model to compare baseline vs. back to baseline (Exp. 1) and baseline vs. drug (Exp. 1 and 2). Cage was the statistical unit for tests of cage type, and rat was the statistical unit for tests of drug treatment and the interaction of drug treatment and cage type. To account for periods when rats were out of sight, we computed mean frequencies and durations per minute. For data presentation purposes, mean durations per min were converted into percent time. All *p* values are two-tailed.

## Results

### Pilot Study

**Fig 2:**
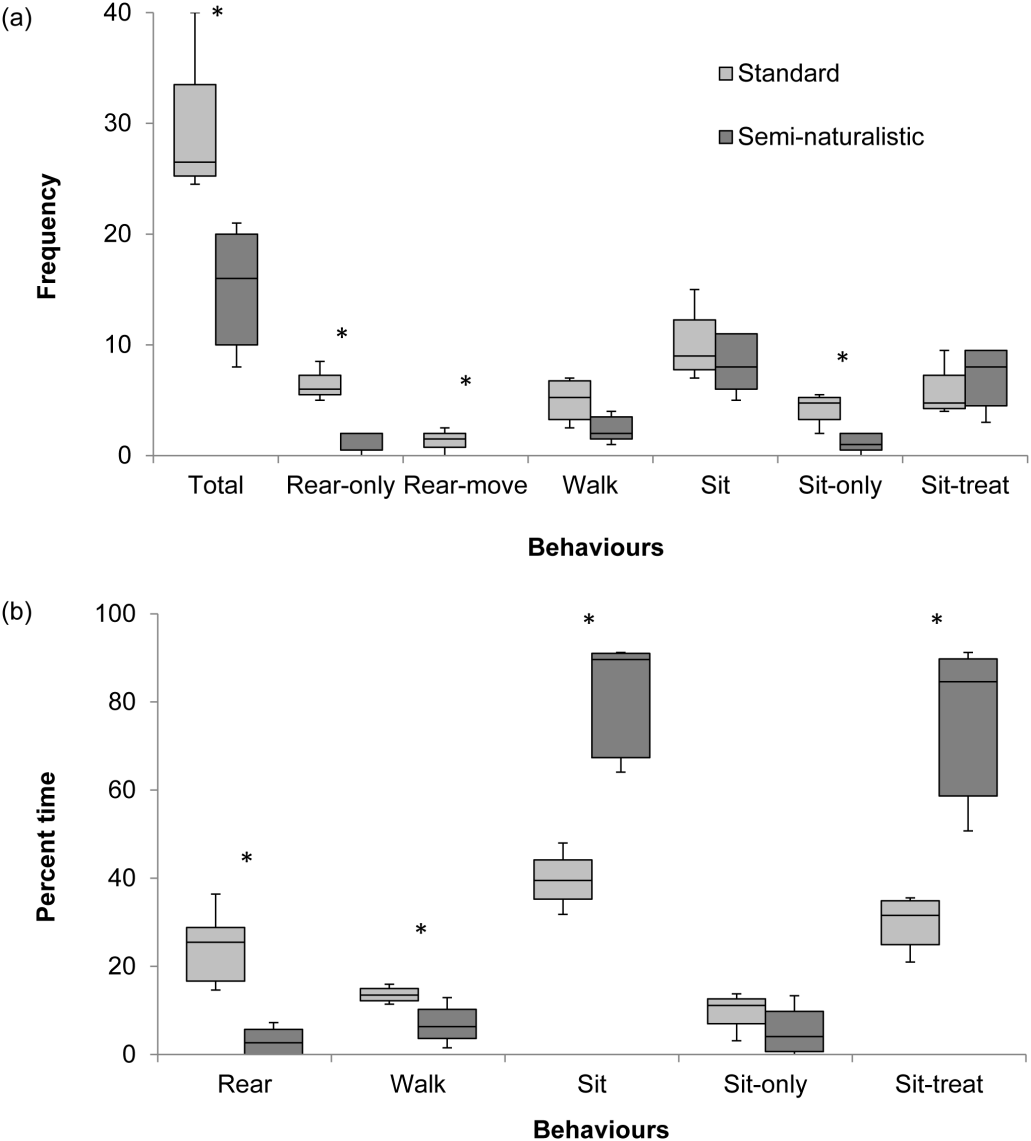
Frequency (a) and percent time (b) of behavioural elements displayed in the Pilot Study. Data presented as medians with 1^st^ and 3^rd^ quartiles as lower and upper limits of the box, and whiskers as lowest and highest data values;; n = 4 standard cages and n = 5 semi-naturalistic cages; *p<0.05.

The total frequency of behaviours was higher in the standard treatment compared to the semi-naturalistic treatment (Fig 2; Z = 2.327; *p* = 0.02). Standard-housed rats performed rear-only and rear-move more frequently (Z = 2.3771; *p* = 0.0175; Z = 2.0494; *p* = 0.0404, respectively) and also spent more time rearing (Z = 2.327; *p* = 0.02). Standard-housed rats also performed sit-only (facing away from the tube) more frequently (Z = -2.1268; *p* = 0.0334) and spent less time sitting (Z = -2.327; *p* = 0.02) and performing sit-treat (with their head in the tube; Z = -2.327; *p* = 0.02). Standard-housed rats spent more time walking (Z = 2.0821; *p* = 0.0373).

### Experiments 1 and 2

Drug treatment and the interaction of drug treatment and cage type had no effect on the total behavioural frequency nor the frequency or duration of any individual behaviours.

**Fig 3:**
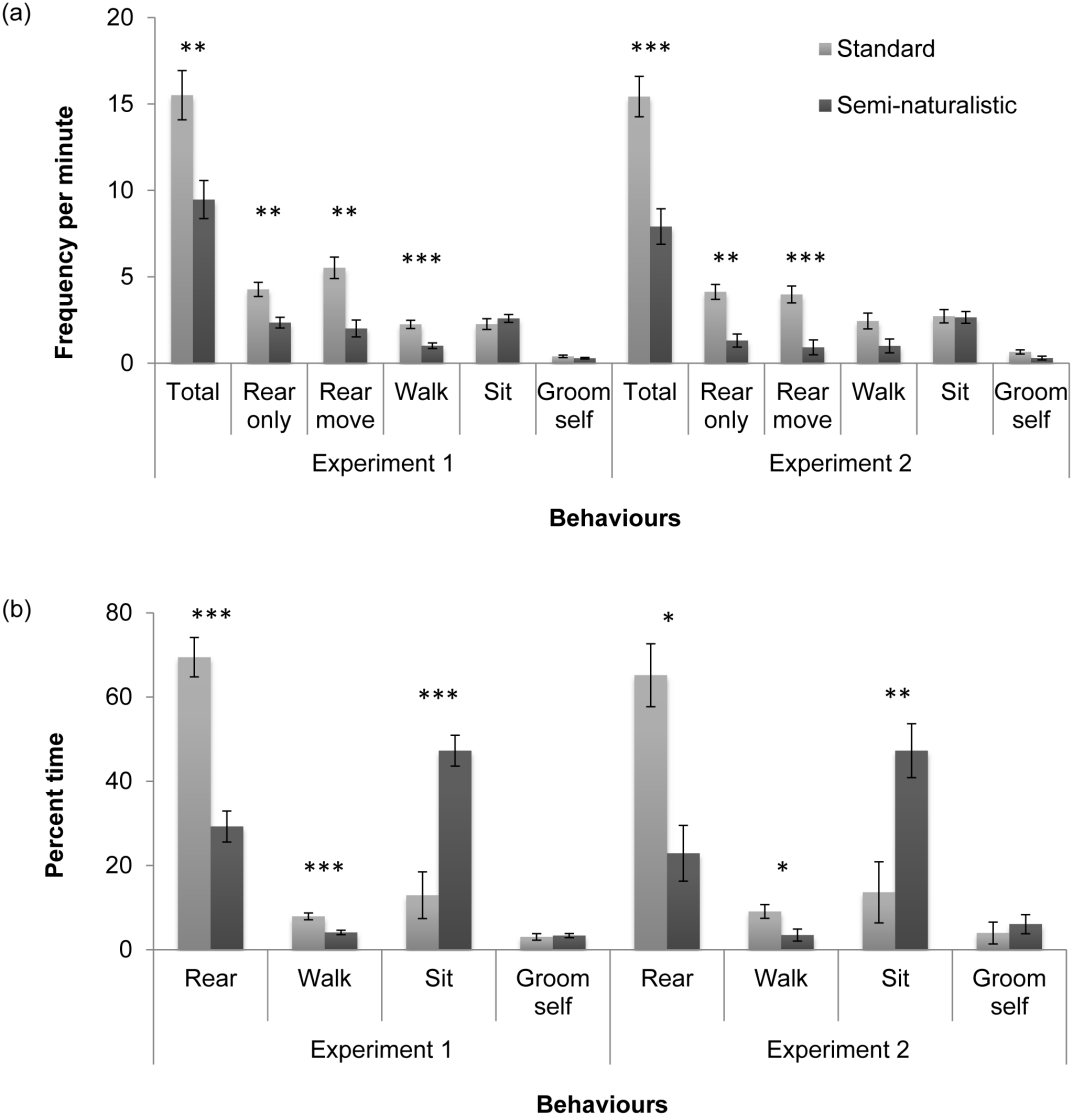
Frequency per minute (a) and percent time (b) for behavioural elements displayed in Experiments 1 and 2. Bars represent LS means ± SEM. In Experiment 1, n = 4 standard cages and n = 6 semi-naturalistic cages, and in Experiment 2, n = 4 standard cages and n = 5 semi-naturalistic cages. Asterisks denote significant differences between the two housing conditions, where *p<0.05; **p<0.01 and ***p<0.001.

In both experiments, the total frequency of behaviours was higher in standard-housed compared to semi-naturalistic-housed rats (Fig 3; Exp. 1: F = 11.49; *p* = 0.0066; Exp. 2: F = 23.2; *p* = 0.0006). In Experiment 1, standard-housed rats performed rear-only (F = 13.89, *p* = 0.0036), rear-move (F = 19.60; *p* = 0.0033) and walk (F = 18.67, *p* = 0.0002) more frequently than semi-naturalistic-housed rats. In Experiment 2, standard-housed rats performed rear-only (F = 24.01; *p* = 0.002) and rear-move (F = 22.31; *p* = 0.0003) more frequently, and tended to walk more frequently (F = 5.53; *p* = 0.0504). In both experiments, standard-housed rats spent more time rearing (Exp. 1: F = 44.51, *p* = 0.0002; Exp. 2: F = 18.77; *p* = 0.0117) and walking (Exp. 1: F = 15.64, *p* = 0.0006; Exp. 2: F = 6.32, *p* = 0.0389) and less time sitting (Exp. 1: F = 26.37, *p* < 0.0001; Exp. 2: F = 14.47, *p* = 0.0019) compared to semi-naturalistic-housed rats.

In general, standard-housed rats tended to rear on one side of the cage, shift positions several times while rearing, sit down briefly before walking over to the other side of the cage, and repeat the sequence. In contrast, rats in the semi-naturalistic environment typically ran to the location of the upcoming treat and sat down, occasionally rearing or sniffing the air before resuming the sitting position. The mean frequency and percent trial time per minute for all behaviours displayed during the anticipatory period in Experiments 1 and 2 are presented in Tables 4 and 5, respectively.

**Table 4:** Frequency per minute and percent time for behavioural elements displayed in Experiment 1.

	Standard			Semi-naturalistic		
	baseline	antidepressant	back to baseline	baseline	antidepressant	back to baseline
**Frequency**						
Agonistic behaviour	0.00±0.01	0.00±0.01	0.00±0.01	0.01±0.01	0.00±0.01	0.02±0.01
Alert	0.00±0.06	0.25±0.06	0.00±0.06	0.06±0.04	0.11±0.04	0.1±0.04
Bite	0.60±0.42	0.11±0.42	0.27±0.42	0.46±0.28	0.46±0.28	0.55±0.28
Climb	n/a	n/a	n/a	0.08±0.05	0.19±0.05	0.22±0.05
Dig	0.00±0.20	0.00±0.20	0.00±0.20	0.10±0.13	0.12±0.13	0.14±0.13
Drink	0.00±0.03	0.06±0.03	0.04±0.03	0.02±0.02	0.01±0.02	0.01±0.02
Eat	0.06±0.04	0.05±0.04	0.12±0.05	0.02±0.03	0.02±0.03	0.00±0.03
Groom self	0.31±0.11	0.45±0.11	0.43±0.11	0.30±0.07	0.28±0.07	0.28±0.07
Groom (social)	0.00±0.04	0.03±0.04	0.00±0.04	0.03±0.03	0.03±0.03	0.06±0.03
Jump	n/a	n/a	n/a	0.04±0.20	0.43±0.20	0.04±0.20
Lie down	0.00±0.01	0.00±0.01	0.01±0.02	0.02±0.01	0.02±0.01	0.01±0.01
Mounting	0.00±0.01	0.04±0.01	0.00±0.01	0.00±0.01	0.01±0.01	0.01±0.01
Rear-only	4.01±0.52	3.78±0.52	5.03±0.55	2.36±0.38	2.43±0.38	2.26±0.38
Rear-move	5.81±0.75	4.83±0.75	5.93±0.79	2.26±0.57	2.07±0.57	1.71±0.57
Shake	0.00±0.01	0.04±0.01	0.00±0.01	0.01±0.01	0.01±0.01	0.01±0.01
Sit	1.88±0.41	2.29±0.41	2.60±0.44	2.65±0.30	2.85±0.30	2.27±0.30
Sit-only	n/a	n/a	n/a	0.48±0.19	0.60±0.19	0.25±0.19
Sit-treat	n/a	n/a	n/a	2.17±0.22	2.24±0.22	2.01±0.22
Sniff (non-social)	0.25±0.11	0.39±0.11	0.12±0.12	0.27±0.08	0.31±0.08	0.14±0.08
Sniff (social)	0.00±0.02	0.03±0.02	0.01±0.02	0.01±0.01	0.02±0.01	0.01±0.01
Stretch	0.00±0.01	0.00±0.01	0.01±0.01	0.00±0.00	0.01±0.00	0.00±0.00
Sway	0.00±0.01	0.01±0.01	0.00±0.01	0.00±0.00	0.00±0.00	0.01±0.00
Walk	2.34±0.33	1.93±0.33	2.50±0.36	1.31±0.22	1.07±0.22	0.64±0.22
Yawn	0.03±0.02	0.00±0.02	0.00±0.02	0.02±0.01	0.01±0.01	0.00±0.01
Total	15.38±1.74	14.09±1.74	17.06±1.83	10.05±1.26	9.87±1.26	8.48±1.26
**Percent time**						
Agonistic behaviour	0.00±0.38	0.00±0.38	0.00±0.41	0.03±0.26	0.00±0.26	0.45±0.26
Alert	0.00±0.41	0.04±0.41	0.00±0.44	0.68±0.30	0.81±0.30	0.61±0.30
Bite	6.96±2.63	1.60±0.96	3.19±1.85	6.05±2.94	4.43±2.94	5.76±2.94
Climb	n/a	n/a	n/a	2.04±0.30	3.82±1.30	4.14±1.30
Dig	0.00±1.27	0.00±1.27	0.00±1.28	0.54±0.54	1.27±1.27	1.28±1.28
Drink	0.00±0.69	0.74±0.69	0.10±0.74	0.87±0.50	0.35±0.50	0.45±0.50
Eat	0.40±0.76	0.38±0.76	0.71±0.82	0.90±0.52	0.37±0.52	0.05±0.52
Groom self	3.08±1.17	3.66±1.17	2.38±1.25	2.88±0.78	3.22±0.78	3.94±0.78
Groom (social)	0.00±1.20	0.18±1.20	0.00±1.29	1.69±0.80	0.57±0.80	1.02±0.80
Jump	n/a	n/a	n/a	0.68±0.30	0.26±0.30	0.03±0.30
Lying	0.00±2.04	0.00±2.04	0.04±2.20	2.87±1.36	0.19±1.36	0.02±1.36
Mounting	0.00±0.24	0.00±0.24	0.00±0.26	0.00±0.16	0.33±0.16	0.00±0.16
Rear	74.03±5.62	65.78±5.62	68.58±5.85	29.13±4.25	29.52±4.25	29.14±4.25
Shake	0.00±0.02	0.07±0.02	0.00±0.02	0.00±0.01	0.00±0.01	0.00±0.01
Sit	6.34±7.08	17.28±7.08	15.17±7.46	44.35±4.72	48.11±4.72	49.33±4.72
Sit-only	n/a	n/a	n/a	11.75±4.38	10.33±4.38	5.73±4.38
Sit-treat	n/a	n/a	n/a	33.47±4.19	38.64±4.19	44.46±4.19
Sniff (non-social)	1.38±0.88	2.10±0.88	0.61±0.93	1.83±0.59	2.56±0.59	1.16±0.59
Sniff (social)	0.00±0.06	0.05±0.06	0.02±0.07	0.01±0.04	0.13±0.04	0.02±0.04
Stretch	0.00±0.03	0.00±0.03	0.09±0.03	0.00±0.02	0.02±0.02	0.00±0.02
Sway	0.00±0.04	0.03±0.04	0.00±0.05	0.00±0.03	0.00±0.03	0.05±0.03
Walk	7.51±0.65	7.84±0.91	8.42±1.26	5.38±0.78	4.21±0.78	2.73±0.78
Yawn	0.06±0.05	0.00±0.05	0.00±0.05	0.07±0.03	0.01±0.03	0.00±0.03

Data are displayed as LS means ± SEM. Individual rats were tested in their home cages in the presence of their cage-mates at baseline and under the influence of an antidepressant. The symbol n/a denotes behaviours that were not possible or not scored in that system; n = 8 rats from four standard cages and n = 18 rats from six semi-naturalistic cages.

**Table 5:** Frequency per minute and percent time for behavioural elements displayed in Experiment 2.

	Standard		Semi-naturalistic	
	baseline	anxiolytic	baseline	anxiolytic
**Frequency**				
Agonistic behaviour	0.00±0.03	0.03±0.00	0.04±0.02	0.00±0.02
Alert	0.00±0.06	0.00±0.06	0.23±0.06	0.12±0.06
Bite	0.11±0.27	0.23±0.27	0.40±0.24	0.00±0.24
Climb	n/a	n/a	0.07±0.03	0.02±0.03
Dig	0.03±0.01	0.00±0.01	0.00±0.01	0.00±0.01
Eat	0.09±0.05	0.08±0.05	0.05±0.04	0.00±0.04
Groom self	0.71±0.17	0.60±0.17	0.35±0.15	0.25±0.15
Groom (social)	0.03±0.06	0.00±0.06	0.04±0.05	0.06±0.05
Jump	n/a	n/a	0.09±0.03	0.00±0.03
Lie down	0.03±0.07	0.00±0.07	0.04±0.06	0.13±0.06
Rear-only	3.77±0.59	4.50±0.59	1.67±0.52	0.95±0.52
Rear-move	4.09±0.60	3.87±0.60	0.97±0.53	0.87±0.53
Shake	0.14±0.05	0.06±0.05	0.02±0.04	0.07±0.04
Sit	2.34±0.60	3.10±0.60	2.58±0.53	2.71±0.53
Sit-only	n/a	n/a	0.54±0.45	0.21±0.45
Sit-treat	n/a	n/a	2.03±0.40	2.49±0.40
Sniff (non-social)	0.51±0.27	1.29±0.27	0.99±0.24	0.65±0.24
Sniff (social)	0.06±0.04	0.06±0.04	0.07±0.03	0.00±0.03
Sway	0.03±0.02	0.00±0.03	0.04±0.03	0.00±0.03
Turn	0.03±0.04	0.06±0.04	0.02±0.04	0.16±0.04
Walk	2.26±0.60	2.62±0.60	0.85±0.53	1.16±0.53
Yawn	0.00±0.04	0.00±0.04	0.00±0.04	0.07±0.04
Total	13.93±1.24	15.81±3.52	7.86±1.72	7.70±1.22
**Percent time**				
Agonistic behaviour	0.00±0.06	0.00±0.06	0.10±0.06	0.00±0.06
Alert	0.00±0.25	0.00±0.25	0.76±0.22	0.46±0.22
Bite	0.24±4.20	1.10±4.30	7.03±3.79	0.00±3.79
Climb	n/a	n/a	0.80±0.32	0.26±0.32
Dig	0.05±0.02	0.00±0.02	0.00±0.02	0.00±0.02
Eat	0.71±2.43	2.28±2.43	2.53±2.15	2.73±2.15
Groom self	4.23±3.02	3.71±3.02	5.17±2.66	6.97±2.66
Groom (social)	0.18±1.35	0.00±1.35	0.49±1.20	1.89±1.20
Jump	n/a	n/a	0.08±0.02	0.00±0.02
Lying	0.55±9.30	0.03±9.30	14.65±8.28	7.47±8.28
Rear	69.61±8.95	60.74±8.95	20.73±7.91	22.07±7.92
Shake	0.08±0.05	0.05±0.05	0.02±0.05	0.11±0.05
Sit	11.66±8.81	15.58±8.81	42.15±7.77	52.37±7.77
Sit-only	n/a	n/a	3.77±4.71	2.58±4.71
Sit-treat	n/a	n/a	38.11±7.27	49.53±7.27
Sniff (non-social)	1.95±1.19	5.26±1.19	3.757±1.05	1.07±1.05
Sniff (social)	0.21±0.12	0.09±0.12	0.19±0.10	0.00±0.10
Sway	0.63±0.39	0.10±0.39	0.38±0.34	0.01±0.34
Turn	0.13±0.21	0.15±0.21	0.04±0.18	0.49±0.18
Walk	8.46±2.07	9.69±2.07	3.13±1.83	3.81±1.83
Yawn	0.00±0.10	0.00±0.10	0.00±0.09	0.17±0.09

Data are displayed as LS means ± SEM. Individual rats were tested in their home cages in the presence of their cage-mates at baseline and under the influence of an anxiolytic. The symbol n/a denotes behaviours that were not possible or not scored in that system; n = 7 rats from four standard cages and n = 9 rats from five semi-naturalistic cages.

## Discussion

These experiments assessed differences in anticipatory behaviour between female Sprague-Dawley rats reared and housed in common standard laboratory cages versus semi-naturalistic environments for more than one year. In all experiments, standard-housed rats were more active while anticipating a reward. Similarly, van der Harst et al. [26] showed that standard-housed male Wistar rats were more active in anticipation of a reward than enriched-housed rats. Our results are consistent with the idea that standard-housed rats are more sensitive to rewards, and suggest that standard-housed rats were experiencing poorer welfare (see [27]) than rats reared in the semi-naturalistic environment.

In Experiments 1 and 2, rats were tested in their home cages and in the presence of their cage-mates; these experiments were primarily designed to test within-rat differences in response to treatment with an antidepressant or anxiolytic. The amount of space and number of cage-mates differed between the two housing treatments; these factors may have encouraged higher activity in the semi-naturalistic cages where it was possible to perform a wider range of behaviours and to make use of a larger area [53], and where there was greater probability of social modulation of behaviour. However, we actually found that rats in the semi-naturalistic environment were less active during the anticipatory period than were the standard-housed rats. This finding is consistent with our results from the Pilot Study, in which rats were tested individually and in a testing arena similar in size for both housing conditions. Our results are not likely explained by cognitive differences caused by markedly different rearing environments, because van der Harst et al. [26] observed similar differences after differentially housing post-pubescent rats for only two weeks. The confound between housing treatment and group size was intentional; we considered a larger group size to be an essential component of the semi-naturalistic environment [2,54]. Further work will be required to determine which specific differences in the housing systems are responsible for the various differences we described.

It would have been helpful to compare differences in the level of activity during the cue-reward interval for rats in each housing treatment before and after anticipatory training. This way, we could have assessed not only absolute differences between rats from different housing conditions, but also changes in each group from baseline. Unfortunately, because rats in the Pilot Study had to be trained extensively to cross the tunnel and enter the treat cage before they could be trained on the anticipatory behaviour task, at the beginning of anticipatory training they already knew to expect a reward in the treat cage. In reality, the purpose of anticipatory behaviour training was likely more to habituate rats to waiting for 60 s rather than to associate cue with reward. Connecting the treat cage (standard-housed rats) or entering the inverted cage (semi-naturalistic-housed rats) were also cues rats likely associated with the upcoming reward. The sound cue used in Experiments 1 and 2 was the same as in the Pilot Study; consequently, rats already associated the cue with a reward and therefore baseline pre-training levels in Experiments 1 and 2 were also unreliable.

This study also showed that the patterns of behaviour–*what* rats did in anticipation of the reward–were different between the two housing treatments. In Experiments 1 and 2, the standard-housed rats spent the most time rearing (Fig 3; approximately 65–70% trial time vs. 20–30% for rats in the semi-naturalistic condition) while semi-naturalistic-housed rats spent the most time sitting (47% vs. 13% for standard-housed rats). However, 75–95% of sitting time in semi-naturalistic-housed rats was spent facing the location of the upcoming treat (see Tables 4 and 5). This suggests that semi-naturalistic-housed rats did anticipate the treat, even though they expressed their anticipation differently from standard-housed rats.

In Experiments 1 and 2 the test cage was different between the two housing treatments; standard-housed rats had to rear to better access smells from the room, while semi-naturalistic-housed rats’ cage was entirely made of bars so room smells were accessible from the sitting position. This difference could explain why standard-housed rats primarily reared while semi-naturalistic-housed rats primarily sat. However, in the Pilot Study all rats were tested in an enclosed ‘standard’ cage (for standard-housed rats, the filter top was on during testing) so rats from both housing treatments could best access room smells through the short tunnel that led to the open treat cage. In this experiment rats from both housing treatments spent three to four times as much time sitting compared to Experiments 1 and 2, but standard-housed rats still spent less time sitting with their head in the tube (sit-treat) and more time walking and rearing. Therefore, differences in what rats did were likely not only due to environmental conditions during testing.

One interpretation for why rats from the two housing conditions behaved differently is that standard-housed rats are more impulsive. Other evidence suggests that rats reared in isolation are more impulsive than environmentally enriched rats. For example, in impulsive choice studies, isolated rats tend to choose smaller, but more immediate rewards, over larger, but delayed rewards [55,56]. A study by Wood and colleagues [57] showed that isolated rats were more impulsive in an operant-shaping procedure in which they would gain access to sucrose by nose-poking a lit hole following a fixed intertrial interval (ITI). The authors found that isolated rats impulsively responded to the operant stimulus by initiating more pokes during the ITI, even though general activity levels were similar between the two groups. The authors argued that isolated rats were more impulsive because they were more sensitive to rewards: since rewards were more salient to them, they had a stronger impulse to seek them. In contrast, a study by Kirkpatrick and colleagues [55] found that isolated rats were less impulsive than enriched-housed rats, but also explained their results in terms of reward sensitivity. The task in Kirkpatrick et al.’s study was similar to the one just described [57], except that responding during the ITI caused the ITI to be reset. With an imposed cost to impulsive responding (longer ITI), isolated rats were actually less impulsive, requiring fewer responses per reward than enriched-housed rats. The authors speculated that isolated rats were more sensitive to rewards, leading them to be less impulsive because this was the most efficient strategy (i.e. in this way they earned the most rewards). Overall, the consensus in the literature seems to be that impulsivity is driven by reward sensitivity. Therefore, both how active rats are and what they do may reflect differences in reward sensitivity. An alternate explanation is that rats reared in restricted environments appear more impulsive because they have little experience with exerting control over, or receiving feedback from, their environment, and therefore failed to learn to inhibit or vary their behaviour in response to external cues (Sackett, 1970, as cited by [58]).

The three most frequent behaviours displayed by standard-housed rats when they were tested in their home cage (Experiments 1 and 2) were rearing, sitting and walking, respectively. This result is somewhat consistent with van der Harst et al. [39] who tested anticipatory behaviour in the home cage of male standard-housed rats. The authors reported that the most frequent behavioural categories displayed by their rats were exploration, arousal and locomotion, where exploration included mobile and immobile exploration and rearing; arousal included running; and locomotion included walking, running and mobile exploration. We did not differentiate between walking and running (running was not possible in our standard cage) and between walking and mobile exploration. The main difference between our findings and theirs is that the second most frequent behaviour displayed by our rats was sitting, while in their study, resting (including sitting) was one of the least frequent behaviours. One factor that could account for the difference is that our rats were tested in the presence of conspecifics, while van der Harst et al. [39] tested animals in the absence of conspecifics. In a separate study that recorded total behavioural frequency but not the frequency of individual behaviours, van der Harst et al. [26] found that rats were more active in the absence of conspecifics, although their rats were also being tested in a different context than they were trained in, so the effects of absence of conspecifics and novel context were confounded. Our rats were also older than rats tested by van der Harst et al. [39], and propensity to sit may increase with age.

Experiments 1 and 2 were also designed to test whether rats were depressed or anxious, by testing their anticipatory behaviour at baseline versus under the influence of an antidepressant or an anxiolytic, respectively. The hypothesis was that if rats were experiencing one of these states, they would exhibit different behaviour when they were given the drug. We found no differences in how rats behaved before and after drug intervention, but regardless of drug treatment, standard-housed rats behaved differently from semi-naturalistic-housed rats. One potential explanation for the lack of difference was the lack of statistical power due to relatively small sample sizes. This explanation seems unlikely, as Experiments 1 and 2 were designed to provide sensitive, within-rat tests of the effect of drug (despite no differences being found) and a weak, between-cage test of cage type (despite many differences being found). Post-hoc power analysis indicated that unreasonably large sample sizes would be required to detect differences with the variance and treatment differences observed (e.g. between 79–1380 rats would have been needed to detect drug effects for rats in the semi-naturalistic condition).

It is also possible that the lack of drug effect suggests that: 1) standard-housed rats were not experiencing conditions analogous to depression or anxiety, even though they are more sensitive to rewards, or 2) our drug interventions were ineffective. In the former case, it is possible that while standard-housed rats were experiencing negative affect compared to semi-naturalistic-housed rats, this negative affect was not analogous to depression or anxiety. Also, the very act of repeatedly announcing a reward is enriching and could have reversed behavioural and neurological effects of a restrictive environment [59,60]. This may have been even truer in the case of the anxiolytic (Exp. 2) because it was tested second.

In the latter case, it is possible that higher doses were needed to successfully treat the severity of depression or anxiety rats were experiencing. For example, the dose of α-S1 tryptic casein given in this study was comparable to a moderate dose of a benzodiazepine [46], but perhaps doses comparable to a high dose of a benzodiazepine were needed. It is also possible that the negative affect experienced by standard-housed rats was not associated with a deficit in the receptors and/or neurons targeted by ketamine and α-S1 tryptic casein; the former likely targets glutamate receptors and GABAergic interneurons, while the latter targets GABA_A_ receptors [40,47]. Finally, it could also be that the drugs did improve affective state, but that these changes in affect do not influence anticipatory behaviour. Von Frijtag et al. [29,30] found that treatment with antidepressants re-established anticipation of a reward in anhedonic rats. Treating anhedonia, in which rats are essentially de-sensitized and unable to interact with the environment, may be different from treating states in which rats are sensitized to rewards; this difference could explain why Von Frijtag et al. [29,30] found that antidepressants modified anticipatory behaviour while we did not.

## Conclusion

Standard-housed laboratory rats are more sensitive to rewards than rats housed in semi-naturalistic conditions, as reflected by the quantity and form of their anticipatory behaviour. This study adds to mounting evidence that standard laboratory housing for rats compromises rat welfare, and provides further scientific support for recommendations that current minimum standards be raised.
